# Feasibility of Extracting Meaningful Patient Centered Outcomes From the Electronic Health Record Following Critical Illness in the Elderly

**DOI:** 10.3389/fmed.2022.826169

**Published:** 2022-06-06

**Authors:** Sumera R. Ahmad, Alex D. Tarabochia, Luann Budahn, Allison M. Lemahieu, Brenda Anderson, Kirtivardhan Vashistha, Lioudmila Karnatovskaia, Ognjen Gajic

**Affiliations:** ^1^Division of Pulmonary and Critical Care Medicine, Mayo Clinic, Rochester, MN, United States; ^2^Department of Internal Medicine, Mayo Clinic, Rochester, MN, United States; ^3^Anesthesia and Critical Care Research Unit, Mayo Clinic, Rochester, MN, United States; ^4^Department of Quantitative Health Sciences, Mayo Clinic, Rochester, MN, United States; ^5^Department of Infectious Disease, Multi-disciplinary Epidemiology and Translational Research in Intensive Care Research Group, Mayo Clinic, Rochester, MN, United States

**Keywords:** critical illness, trajectory, patient important outcomes, days alive and out of hospital/health care facility (DAOH), electronic health records (EHR)

## Abstract

**Background:**

Meaningful patient centered outcomes of critical illness such as functional status, cognition and mental health are studied using validated measurement tools that may often be impractical outside the research setting. The Electronic health record (EHR) contains a plethora of information pertaining to these domains. We sought to determine how feasible and reliable it is to assess meaningful patient centered outcomes from the EHR.

**Methods:**

Two independent investigators reviewed EHR of a random sample of ICU patients looking at documented assessments of trajectory of functional status, cognition, and mental health. Cohen's kappa was used to measure agreement between 2 reviewers. Post ICU health in these domains 12 month after admission was compared to pre- ICU health in the 12 months prior to assess qualitatively whether a patient's condition was “better,” “unchanged” or “worse.” Days alive and out of hospital/health care facility was a secondary outcome.

**Results:**

Thirty six of the 41 randomly selected patients (88%) survived critical illness. EHR contained sufficient information to determine the difference in health status before and after critical illness in most survivors (86%). Decline in functional status (36%), cognition (11%), and mental health (11%) following ICU admission was observed compared to premorbid baseline. Agreement between reviewers was excellent (kappa ranging from 0.966 to 1). Eighteen patients (44%) remained home after discharge from hospital and rehabilitation during the 12- month follow up.

**Conclusion:**

We demonstrated the feasibility and reliability of assessing the trajectory of changes in functional status, cognition, and selected mental health outcomes from EHR of critically ill patients. If validated in a larger, representative sample, these outcomes could be used alongside survival in quality improvement studies and pragmatic clinical trials.

## Introduction

The measurement of important outcomes of critical illness continues to evolve. Medical advances have resulted in significant improvement in mortality despite increasing age, severity of illness, and number of comorbidities (1, 2). Most randomized controlled trials in critical care research are negative studies, with problems pertaining not just to statistical analysis and heterogenous biological attributes but also the choice of “mortality” as a measurable but crude outcome of critical illness (3, 4). Mortality is just one side of the coin, since challenges of critical illness survivorship come at a cost of pain, anxiety, depression, impaired cognition, and debility, collectively described as post-ICU syndrome (PICS)(5, 6). Amongst the most relevant predictors of outcomes of survival are older age, comorbidities, and pre- ICU health status (7–9).

Significant effort has been dedicated toward identifying the most relevant domains by which clinicians and scientists can measure outcomes meaningful to patients. About 250 tools have been used in clinical research to capture relevant health domains hindering comparison of results (10). Measurement properties of such tools per consensus-based standards are inadequately assessed for in studies reporting them (11). The National Institute of Health (NIH) has developed a framework of key components of patient reported outcomes that can be used in clinical studies (12–16) to help address survivorship. Health trajectories comparing baseline/ pre- ICU status to post ICU follow ups have been accomplished in individual domains such as cognitive status, functional status or as health-related quality of life (HRQOL) using some of these measurement tools (17–21). Today, survival, HRQOL, pain, cognitive function, physical function, mental health, muscle/nerve function and pulmonary function have been identified as the most salient domains from the standpoint of clinicians, researchers, and patients and their families (12–16).

Days alive and out of hospital (DAOH), also known as hospital free days has also gained increased interest in studies as a pragmatic patient centered outcome (22). It has been captured in larger registries in the frail population and after hip fracture (23, 24). Elderly patients have expressed, that time spent at home, especially near end of life, as a very important preference (25).

Administration of validated measurement tools can be time consuming, costly, and impractical outside large research settings. A previous comparison between health survey and EHR of outcomes of multimorbidity showed that both methods were equivalent in the elderly group (26). No study has evaluated the quantity and quality of EHR representation of patient centered outcomes when approaching a combination of outcomes of functional status, cognitive status and mental health, or the trajectory of these outcomes before and after critical illness.

We therefore hypothesized that we could abstract variables from “free text” contained in the EHR which could provide valuable insight into the pre to post ICU trajectory in these domains. We chose DAOH to represent days alive and out of hospital/ health care facility, as a complimentary meaningful outcome to assess from EHR. If feasible, EHR assessments could be automated to provide a scalable, clinically useful method for studying meaningful patient centered outcomes in pragmatic trials and quality improvement interventions.

## Methods

### Study Design

**Figure 1:**
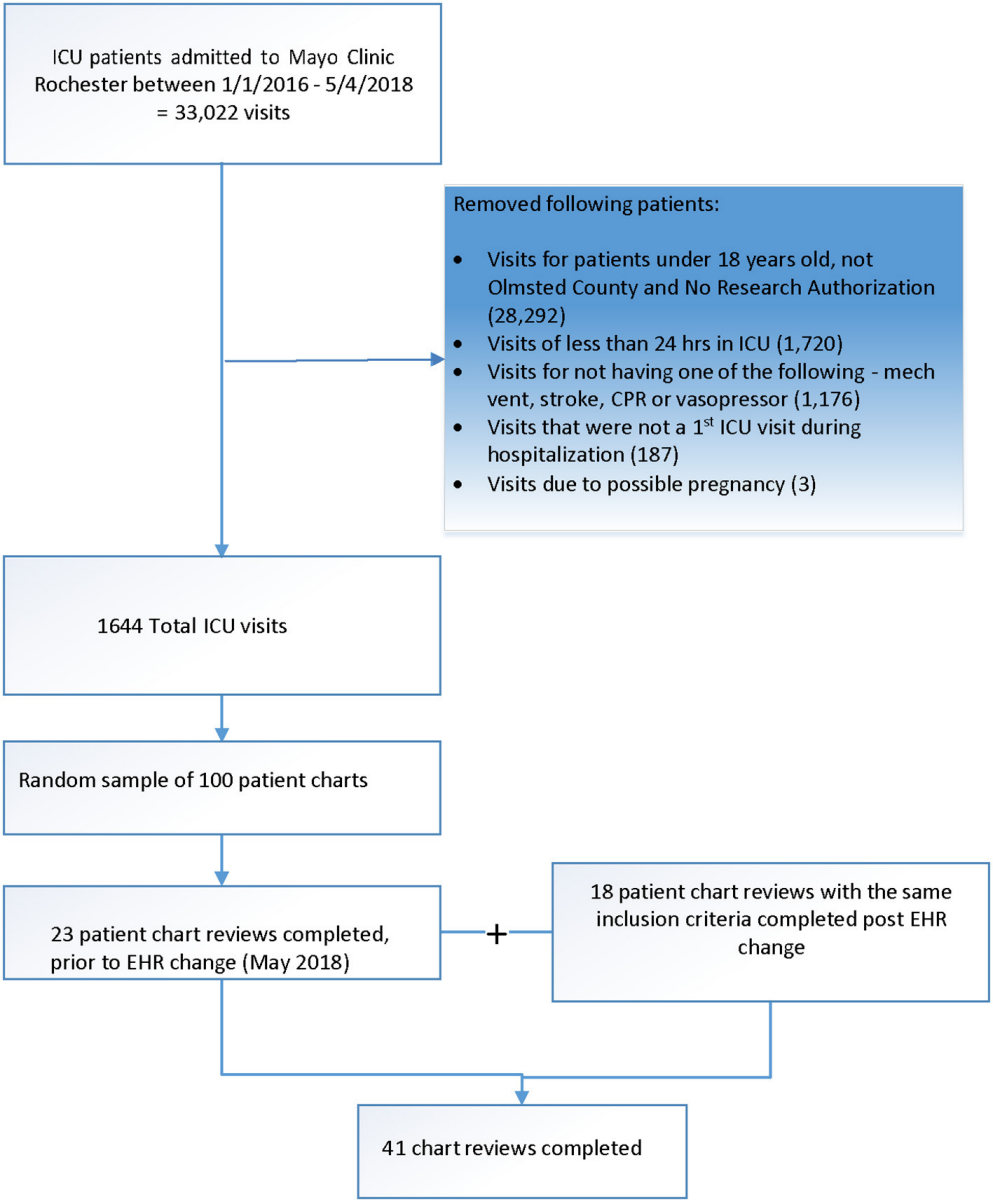
Consort diagram of patient chart review.

This is a feasibility study comprising of a single center retrospective review of the EHRs in a random sample of elderly ICU patients between 2015 and 2019 at the Mayo Clinic, Rochester, MN. The study was approved by the Mayo Clinic Institutional Review Board (#18-004302). The institutional EHR was changed to Epic in May of 2018 and patient information documented prior to 2018 was also uploaded into Epic (Figure 1). The Anesthesia and Critical Care Research Unit (ACRU) at the Mayo Clinic assisted with data extraction and manual chart review; latter was performed by SA and LB.

Data on demographics, diagnoses, hospital outcomes and patient related outcomes including functional status, cognition, and mental health was stored in a REDCap data base. The REDCap data collection questions pertaining to patient outcomes were designed using the qualitative technique of data saturation, whereby questions were continually developed and locations for such information were identified during chart review (27). Charts were continually revisited and studied until the relevant content identified by each reviewer was abstracted by both reviewers. The information was then evaluated by each reviewer to determine if trajectory of functional status, cognition, and mental health in the 12-month time frame prior to illness and within 12-months after discharge for survivors could be established to look for meaningful changes in survivors. Discrepancies were identified by each reviewer for resolution during intergroup discussions and when needed by a third reviewer: AT.

Each domain was reviewed for relevant chart documentation with key words used to identify the outcome in the chart (Supplementary Table 1), reflective of aspects of body function and structures, activities and participation described in World Health Organization (WHO) description of International Classification of Function (ICF) in 2001. For this feasibility study, functional status included aspects of physical function and activities of daily living. Some key themes of cognition included that of attention, alertness, and memory. Similarly, mental health included the search for depression, anxiety, and post -traumatic stress disorder (PTSD). For example, functional status could be captured as a diagnosis of “repeated falls” or “frailty”, a description of a specific activity of daily living, or the need of a certain support such as a walking aid. Other parameters included specific medications, validated tools documenting the functional, cognitive, and mental health status and ICD-10 (international classification of diseases) codes. For example, the use or new prescription of antidepressant could help identify encounters discussing depression. Similarly, validated tools such as PHQ (patient health questionnaire)-9 for depression or mental status exam tools can be found in charts as well. Of note, ICD- 10 codes were an automated query and were available for charts abstracted prior to institutional EMR change.

Particular attention was paid to documentation and clinical discourse capturing patient/provider quotes and any descriptions of patient's narratives as they related to the outcome of interest (Supplementary Table 2). For charts where there was inadequate information to support the presence or absence of a particular outcome, we categorized it as “unable to determine”.

Since chart review included 12 months EHR follow up, discharge dates, number of admissions and integrated rehabilitation days were also tracked in the EMR to assess the ability to compute DAOH.

### Participants

Patients over 65 years old admitted to the ICU from 8/23/2015 to 8/7/2019 for >24 h and requiring one or more of the following: high-flow nasal cannula, bilevel or continuous positive pressure ventilation, invasive mechanical ventilation, vasopressor need were selected for potential inclusion to this study. Despite variable age cut offs in the aging population, for the purpose of this study, we defined elderly as age more than 65 years. The patients included in the study were residents of Olmsted County, Minnesota, with research authorization on file. Patients in a vegetative state as baseline or those requiring intubation for elective surgical procedures were excluded. In total, 41 patient chart reviews were completed, with 23 having index ICU admission occur prior to EHR change and 18 after.

### Outcomes

The primary end point was to evaluate whether it is possible to establish the pre- to post-ICU trajectory in the domains of functional status, cognition and mental health using the EHR in a 12-month chart follow up.

The secondary end point was to evaluate whether DAOH, can be extracted from the EHR as a complimentary meaningful patient outcome up to the 12-month EHR follow up.

### Statistical Analysis

Patient characteristics, interventions, and outcomes were summarized using median and interquartile range (IQR) for continuous variables and frequency counts and percentages for nominal variables. Cohen's kappa coefficient was used to measure agreement between the two reviewers regarding change in pre- and post-ICU quality of life (28).

DAOH was considered zero if a patient was unable to be discharged home after short term rehabilitation. Days after ICU admission were calculated, observing for survival post ICU, date of death within the 12-month EHR follow up, hospital discharge day and number of days of recurrent admissions to the hospital. The category of “some days at home” contained a range of calculated DAOH.

Data management and statistical analysis were performed in SAS Studio 3.8 (SAS Institute Inc, Cary, North Carolina) and Microsoft Excel 365 (Microsoft Corporation, Redmond, WA).

## Results

Two independent investigators completed EHR reviews of a random sample of 41 patients (Figure 1). Five (12%) patients died during the hospitalization. Table 1 describes demographic and clinical characteristics of these patients. A variety of note sources were reviewed where pertinent information was found. Change in each of the specific domains of functional status, cognitive status and mental health were ascertained by evaluating a combination of notes before and after critical illness in the EHR. For example, if there was clinical documentation of normal activities of daily living pre-ICU and wheelchair dependence post ICU, that would result in “worsening” functional status. Another example, if there was no cognitive dysfunction in pre-ICU assessment and no concern raised in sequential follow up notes despite adequate documented dialogue, then the impression was “no change”. Where there was insufficient follow up or documentation, we considered those situations as “unable to determine.” Some high yield notes besides routine inpatient and outpatient notes included social work, physical/occupational therapy, nursing and subspecialty notes (Supplementary Table 3). Very detailed pre- ICU functional status, cognition and mental health were best captured in social work notes and physical and occupational therapy notes. Post- ICU, outpatient notes at rehabilitation or clinic were higher yield. Free texts in “chart documentation” were the most common source of information (Supplementary Table 1). Specific medications were most helpful for identifying and exploring mental health domain. Validated tools for each domain were found less commonly in the before and after critical illness time frame, such as in 4 charts for each functional and cognitive status and 7 charts for mental health (Supplementary Table 1).

**Table 1:** Patient characteristics.

**Variable**	**Overall (*N* = 41)**
**Demographics and pre-ICU characteristics**	
Gender, n (%)	
Female	12 (29%)
Male	29 (71%)
Age, median (Q1, Q3)	75.0 (69.0, 84.0)
Pre-ICU residence, *n* (%)	
Assisted living	5 (12%)
Home	30 (73%)
Nursing home	5 (12%)
Short-term nursing facility	1 (2%)
Pre-existing chronic mechanical ventilation, *n* (%)	1 (2%)
Pre-existing tracheostomy, *n* (%)	1 (2%)
Pre-existing feeding tube, *n* (%)	1 (2%)
Chronic hemodialysis, *n* (%)	0 (0%)
Number of medications on at admission, *n* (%)	
<5	5 (12%)
5-10	10 (24%)
11-15	11 (27%)
>15	13 (32%)
Unable to determine	2 (5%)
ICU Interventions for study inclusion	
Mechanical ventilation (new), *n* (%)	33 (80%)
Non-invasive mechanical ventilation (new), *n* (%)	16 (48%)
Vasopressor support, *n* (%)	29 (71%)
Hospital outcomes	
Status at hospital discharge, *n*(%)	
Alive	36 (88%)
Dead	5 (12%)
Hospital length of stay, median (Q1, Q3)	12.0 (7.0, 17.5)
Status at ICU discharge, *n* (%)	
Alive	39 (95%)
Dead	2 (5%)
ICU length of stay, median (Q1, Q3)	5.0 (2.3, 7.0)

The majority (86%) of charts contained sufficient information to determine the pre- ICU to post- ICU health state trajectories (Table 2). There was almost perfect inter-rater agreement between the two reviewers with kappa values of 0.966, 1 and 1 in functional status, cognitive status, and mental health trajectories, respectively.

**Table 2:** Change in pre- and post-ICU functional status, cognition, and mental health, per EHR review with corresponding agreement between two independent reviewers.

**Variable**	**Total survivors (*N* = 36)**	**Kappa**
Change in functional status, *n* (%)		0.966
Better	2 (6%)	
Unchanged	15 (42%), 16 (44%)^*^	
Worse	13 (36%)	
Unable to determine	5 (14%), 4 (11%)^*^	
Change in cognitive status, *n* (%)		1.000
Better	0 (0%)	
Unchanged	26 (72%)	
Worse	4 (11%)	
Unable to determine	5 (14%)	
Change in mental health, *n* (%)		1.000
Better	1 (3%)	
Unchanged	25 (69%)	
Worse	4 (11%)	
Unable to determine	5 (14%)	

* *Represents where Reviewer 1 and Reviewer 2 had a difference in their assessments*.

*The first n (%) value represents Reviewer 1. The second n (%) value represents Reviewer 2*.

Eighteen (44%) of patients remained at home at 12 months after discharge from hospital or short-term rehabilitation, whereas 13 (32%) had zero DAOH, including those who died or ended up in long term health care facility (Table 3). The category “some days at home” contained a range of calculated DAOH between 1 and 288 days.

**Table 3:** Days alive and out of hospital/ health facility (DAOH) after discharge within 12 months per EHR.

**Categories of DAOH**	**Overall (*N* = 41)**
Zero days	13 (32%)
^*^Some days	5 (12%)
^**^Remained at home	18 (44%)
Unable to determine	5 (12%)

* *Some days reflects time out of a health care facility but not at 1-year post discharge*.

** *Remained at home reflects days up to one-year post discharge*.

## Discussion

This pilot study shows that it is feasible to use EHR to extract qualitative change to reveal a trajectory in meaningful patient centered outcomes after critical illness including functional status, cognition, mental health, and days alive and out of hospital/health care facility (DAOH). EHR sources included a variety of multi-professional notes. Notably, social worker and physical and occupational therapy notes were quite comprehensive in capturing a combination of these domains as patient's pre-ICU baseline. There was excellent agreement between two independent reviewers.

A multifaceted approach to the search was needed to explore each domain to adequately assess the health status before and after critical illness rather than rely on any one parameter. The highest yield parameter was free texts. Medications helped identify mental health. Trajectories relying alone on validated tools were inadequate in this exploration. For example, Barthel index may be used in rehabilitation, but not in an outpatient clinical encounter or even in the hospital where another type of tool is used, and hence comparison is difficult.

Despite concerns that EHR contained inadequate documentation to be a reliable source for outcomes of functional status, cognitive status, or mental health (29–32), it has been widely used in clinical research. A review of implementing WHO Internation Classification of Function (ICF) in EHR, revealed benefits of universal applicability of ICF, collaboration and user satisfaction, though also picked on challenges with clinical terminologies and development of ontologies (33).

The Barthel index, one of the validated tools to assess functional status, has been computed using EHR nursing documentation to capture pre-ICU baseline functional status and at 1 year follow up to assess new functional impairments in patients receiving mechanical ventilation (34). A strategy of deriving functional status from documented activities of daily living (ADL) has previously been applied with the help of EHR (35–37). Nursing documentation of mobility of elderly patients identified those at risk for poor outcomes (38). EHR chart review of physical and occupational therapy and discharge notes, revealed association of higher functional dependence in COVID-19 survivors with higher burden of disease and increased age (39). EHR has been successfully used to derive variables using ICD-10 codes to develop frailty risk score (40).

For cognitive function, documentation is more consistent in the EHR for dementia than for milder cognitive impairment (31). Validated test scores for dementia are rarely available in EHR, though algorithms for diagnosis codes and prescriptions for dementia are found to be reliable (41). In contrast, the ability of detecting cognitive impairment with ICD codes and frailty index in annual Medicare wellness checks of survivors of critical illness was very low (42). However, using key terms such as *cognitive deficit, impaired memory, impaired judgement*, to develop an EHR algorithm resulted in excellent sensitivity and specificity to capture cognitive impairment that outperformed ICD codes (43).

No previous EHR study observed mental health outcomes of depression and anxiety in critical illness. Mental health diagnoses of depression and anxiety have been explored with EHR looking at some combination of variables such as rates of psychotropic medications, diagnoses codes, referrals for mental health services and in validated tools such as PHQ-9 (44–46). Documentation for anxiety in critical illness reviewing provider notes was found in about 45% of sampled chart reviews with terms such as *panic, anxiety*, and *distress* (47). Capturing PTSD is particularly challenging. An algorithm for diagnosis of PTSD in EHR has compared very well with chart review (48). However, documentation of PTSD in EHR has been fraught with omission of information resulting in missing data in studies from the Veteran Affairs and primary care (49, 50). In our sample of charts, we did not find any documentation of PTSD.

DAOH has surfaced as a promising pragmatic patient centered outcome but further refinement is required prior to more widespread use (22). There is heterogeneity in the intervals of measurements from hospital discharge or time of randomization to ranges in between 28 days and 365 days (51–55). The accuracy of DAOH calculation from EHR is limited since long term acute care days may be imprecisely accounted for (22), similar to our experience.

Our study differs in several ways. We assess the feasibility of extracting a combination of more than one or two outcomes, provide a methodology of the process, and track a trajectory of pre-ICU to post- ICU health. This identified high yield areas in the EHR that progressively made the process of chart review easier and for the reviewers to form mutual agreement. We employed a multifaceted approach using search for relevant medications, tools, diagnosis code, and chart review with specific documentation in the form of free text. Free text dialogue observed in our study captured a discourse, such as a new description of “*wheelchair bound”* after critical illness to reflect a change in functional status, or “*mood is down and sad”* to support clinical documentation of mental health change. This is similar to how free texts in EHR have been used to map presence of anxiety and depression symptoms, “*easy to wake u”* and “*depressed or sad mood”* (56). This approach could be further developed into an ontology for free text data mining with automated Natural Language Processing (NLP). Such ontologies have been previously built to capture frailty in cardiac patients and functional status in patients with colorectal cancer (57, 58). NLP and machine learning has also been extensively used to in studies for mental health (59, 60). In critical care, NLP has been used to predict outcomes, using discrete clinical free texts such as *sepsis, pupils fixed* and *coagulopathy* (61). NLP for inclusion criteria has also been used for recruiting patients for a randomized clinical trial in sepsis (62).

Several weaknesses limit the interpretation of this feasibility study. First, much of what can be abstracted in the free text depends on the quality and variability of documentation and identifying relevant note sources. Data saturation by two reviewers was used to help minimize this limitation. The substantial documentation of these outcomes encountered may be reflective of our multi- disciplinary approach toward patient care at our institution. Second, chart review is subjective, allowing for reviewers to have unique interpretations of the same data. These discrepancies were resolved within our group discussions. Third, the timeline of this study includes a change in the EHR system and aspects of documentation have changed with refining the EHR since. Fourth, in computing DAOH, documentation on length of stay in rehabilitation facilities was not always accurately observed in the EHR. Fifth, this is a single center feasibility study with a small sample size, and it was not possible to assess the incidence or prevalence of the outcomes, nor was this the intention of the study. Sixth, and perhaps the most important limitation is that the trends in functional status, cognition, and mental health identified from EHR may not always directly compare to validated tools, since one is the impression of the provider, and the tools reflect those of the patient. Both methods certainly offer unique perspectives and can potentially complement each other.

With an increasing focus on post- ICU recovery, improving the efficiency of extracting these domains will be essential. Annotating data, identifying themes and texts to develop automated queries in EHR, could help create algorithms and a NLP ontology (63) to develop ‘meaningful patient centered outcomes’ and for mapping to tools for validation as recommended in *https://www.improvelto.com*. Such an outcome measure if reliable would be ideal for use in pragmatic trials, quality improvement interventions as well as a humanized digital platform for patient care. The ability to identify such outcomes in EHR can help then analyze a chart more efficiently to determine trajectory of health before and after critical illness. DAOH, could also be automated, if rehabilitation encounters are more accurately captured in the EHR. Future directions to this work would include repeating such EHR explorations from academic tertiary centers as well as community hospitals to account for the differences in documentation and content present in EHR. There may be variability in availability of resources, such as physical and occupational therapy and social work to account for differences in EHR content. There is also an opportunity to simplify validated tools for consistent patient interactive use in EHR.

Eventually, the goal is to develop a pragmatic method to measure a combination of meaningful patient centered outcomes complementary to routine outcomes like mortality and health care lengths of stay usual in critical care research.

## Conclusion

The health domains of functional status, cognitive status, mental health, trajectory of pre to post ICU health, and days at home after critical illness are some of the key domains of meaningful patient centered outcomes. It is feasible to observe a trajectory of these outcomes pre-ICU and post-ICU in EHR reviews which could be further developed for use in pragmatic critical care research.

## Data Availability Statement

The original contributions presented in the study are included in the article/Supplementary Material, further inquiries can be directed to the corresponding author/s.

## Ethics Statement

The studies involving human participants were reviewed and approved by Mayo Clinic Institutional Review Board (#18-004302). Written informed consent for participation was not required for this study in accordance with the national legislation and the institutional requirements.

## Author Contributions

SA: conceptualization, methodology, software, investigation, formal analysis, visualization, writing original draft, project administration, and funding acquisition. AT: validation, investigation, and writing original draft. LB: investigation and data curation. AL: formal analysis, visualization, and writing original draft. BA: resources and project administration. KV: methodology and software. LK: resources and writing-review and editing. OG: conceptualization, supervision, and writing-review and editing. All authors contributed to the article and approved the submitted version.

## Funding

This work was supported by Small Grant Award, Mayo Clinic PI SA, FP00100342 and Critical Care Research Subcommittee, Mayo Clinic PI SA, Company 300, PAU 43306.

## Conflict of Interest

The authors declare that the research was conducted in the absence of any commercial or financial relationships that could be construed as a potential conflict of interest.

## Publisher's Note

All claims expressed in this article are solely those of the authors and do not necessarily represent those of their affiliated organizations, or those of the publisher, the editors and the reviewers. Any product that may be evaluated in this article, or claim that may be made by its manufacturer, is not guaranteed or endorsed by the publisher.
