# Supplementary material for: Feasibility of Extracting Meaningful Patient Centered Outcomes From the Electronic Health Record Following Critical Illness in the Elderly
Source: Front Med (Lausanne). 2022 Jun 6;9:826169. doi: 10.3389/fmed.2022.826169 (PMC9207323; doi:10.3389/fmed.2022.826169)
Supplement: Supplementary file 1 [file Table_1.DOCX]

Supplementary Table 1. Framework of EHR extraction of meaningful outcomes pre and post ICU, N= number of charts reviewed with each category

|  | Functional status | Cognitive status | Mental health: depression, anxiety, PTSD |
| --- | --- | --- | --- |
| Chart documentation | N= 39  1 - Slow gait, repeated falls  2 - Walking aid/ wheelchair / personal assistant  3 - Hemiplegia/ quadriplegia/ paresis  4 - Bed- bound  5- Frailty  6 - Documentation of failure to thrive  7 - Other specific documentation  8 - Unable to determine | N=19  1 - Dementia  2 - Cognitive impairment  3 - Memory impairment  4 - Forgetfulness  5 - Orientation  6 - Other specific documentation  7 - Unable to determine | N=21  1-Depression  2-Anxiety  3-PTSD  4-Other  5 - Unable to determine |
| Tools used | N=4  1 - Barthel index  2 - SF 36  3 - EQ-5D-3L  4 - Katz Activities of Daily Living  5 - Lawton's Instrumental Activities of Daily Living  6 - Functional Status Score for Intensive Care Unit (FSS-ICU)  7 - AM-PAC "6-clicks" Basic Mobility  8 - Other tool used | N=4  1, MMSE  2, Trail making tests  3, Cognitive Log (Cog-Log) and/or Orientation Log (O-Log)  4- AM-PAC "6-clicks" Daily Activity  5-Other tool used | N=7  1 - HADS  2 - PHQ-9  3 - Beck's inventory  4 - Impact of event scale (IES)  Other, specify |
| Specific medications | N=0  Not applicable | N=0  1 - Namenda  2 - Donepezil  3 - Sinemet | N=8  1 - Amitriptyline  2 - Nortriptyline  3 - Citalopram  4 - Fluoxetine  5 - Venlafaxine  6 - Duloxetine  7 - Sertraline  8 - Bupropion,  9 - Lithium  10 - Gabapentin  11 - Topiramate  12 - Pregabalin  13 - Xanax  14 - Ativan  15 - Quetiapine  16 - Other medication for mental health |
| Combination of methods | N=9 | N=8 | N=14 |
| ICD10 code  **automated query,*  *Available for 23 patients* | N= 9  36- item list of following themes  1 - Slow gait/ repeated falls  2 - Walking aid/ wheelchair dependent/ personal assistant  3 - Hemiplegia/ quadriplegia/ paresis  4 - Bed bound  5 - Failure to thrive  6 - Frailty | N= 9  37- item list of following themes  1 - Dementia  2 - Cognitive impairment/memory impairment  3 - Disorientation/ delirium | N=7  40 item list of following themes  1 - Depression  2 - Anxiety  3 - PTSD  4 - Adjustment disorder |
