# Supplementary material for: Feasibility of Extracting Meaningful Patient Centered Outcomes From the Electronic Health Record Following Critical Illness in the Elderly
Source: Front Med (Lausanne). 2022 Jun 6;9:826169. doi: 10.3389/fmed.2022.826169 (PMC9207323; doi:10.3389/fmed.2022.826169)
Supplement: Supplementary file 2 [file Table_2.docx]

Supplementary Table 2. Examples of EHR review including use of free text and note sources

|  | Examples of documentation for pre-ICU | Examples of documentation during ICU stay | Examples of documentation for post-ICU | Types of notes |
| --- | --- | --- | --- | --- |
| Functional status | - Preparing meals, feeding themselves, dressing, using the toilet, housekeeping, bathing, walking, using transportation, getting in/out of bed, and managing medications. | - AM-PAC: inpatient short form  Basic Mobility Raw Score: 15  Basic Mobility Standardized Score: 39.45  Patient is currently below her baseline functional level and is requiring assist x 1 for all mobility | - Lives independently in her home independent of ADLs | - SW note  - PT/OT  - Outpatient primary care note |
| Cognitive status | - Symptoms of cognitive decline about 8 months prior to the visit, progressively  - Known Alzheimer’s disease | - Cognition:  Orientation: Oriented X4  Impulsive: Mild  Attention: Impaired  Divided: Difficulty with extraneous information during moderately complex visual scanning tasks.  Memory: Impaired  Following Commands: Follows one step commands without difficulty  Problem Solving: Impaired |  | *- neurology notes*  *- OT* |
| Mental health: depression, anxiety, PTSD | -Depression and generalized anxiety disorder in past year. On sertraline for it | SW note: mental health assessment is normal (questions of suicide, self- harm, hopelessness). Euthymic. Mood congruent.  Anxiety Symptoms: No symptoms of panic  Depressive Symptoms: No symptoms of depressions | - Her mood has been significantly depressed in dealing with these medical concerns. In addition, she lost her husband and lost her home within the past 5 years. | - Progress note  - SW note  - outpatient internal medicine note |

- PT: physical therapy
- OT: Occupational therapy
- SW: Social work
- AM-PAC: Activity Measure for Post-Acute Care, scoring used inpatient to assess functional status
