# Supplementary material for: Feasibility of Extracting Meaningful Patient Centered Outcomes From the Electronic Health Record Following Critical Illness in the Elderly
Source: Front Med (Lausanne). 2022 Jun 6;9:826169. doi: 10.3389/fmed.2022.826169 (PMC9207323; doi:10.3389/fmed.2022.826169)
Supplement: Supplementary file 3 [file Table_3.DOCX]

Supplementary Table 3. Specific note types in EHR

|  | Functional pre-ICU | Cognitive pre-ICU | Mental pre-ICU | Functional post-ICU | Cognitive post-ICU | Mental post-ICU |
| --- | --- | --- | --- | --- | --- | --- |
| Outpatient | 8 (20%) | 8 (20%) | 12 (29%) | 13 (32%) | 3 (7%) | 6 (15%) |
| Inpatient | 8 (20%) | 5 (12%) | 5 (12%) | 10 (24%) | 3 (7%) | 1 (2%) |
| SNF/rehabilitation | 0 (0%) | 3 (7%) | 2 (5%) | 2(5%) | 3 (7%) | 4 (10%) |
| Social work | 26 (63%) | 7 (17%) | 9 (22%) | 3 (7%) | 2 (5%) | 0 (0%) |
| Physical and occupational therapy | 11 (27%) | 3 (7%) | 0 (0%) | 3 (7%) | 0 (0%) | 0 (0%) |
| Subspeciality | 4 (10%) | 2 (5%) | 4 (10%) | 3 (7%) | 1 (2%) | 4 (10%) |
| Generalized chart review | 2 (5%) | 13 (32%) | 8 (20%) | 5 (12%) | 18 (44%) | 13 (32%) |
| Unable to determine | 0 (0%) | 2 (5%) | 7 (17%) | 5 (12%) | 5 (12%) | 6 (15%) |
| NA | 0 (0%) | 0 (0%) | 0 (0%) | 8 (20%) | 8 (20%) | 8 (20%) |

- Inpatient notes include prior or later admissions, discharge summaries, emergency visits
- Generalized chart review: examining a variety of note sources
- SNF: short term rehabilitation
- Unable to determine: if documentation insufficient to conclude from or patient did not follow in the system
- NA: not applicable if the patient died in hospital or shortly after
